# N-3 PUFA Ameliorates the Gut Microbiota, Bile Acid Profiles, and Neuropsychiatric Behaviours in a Rat Model of Geriatric Depression

**DOI:** 10.3390/biomedicines10071594

**Published:** 2022-07-04

**Authors:** Te-Hsuan Tung, Yang-Ching Chen, Ya-Tin Lin, Shih-Yi Huang

**Affiliations:** 1 School of Nutrition and Health Sciences, Taipei Medical University, Taipei 110301, Taiwan; da07107001@tmu.edu.tw (T.-H.T.); melisa26@tmu.edu.tw (Y.-C.C.); 2 Graduate Institute of Metabolism and Obesity Sciences, Taipei Medical University, Taipei 110301, Taiwan; yatinlin@tmu.edu.tw; 3 Department of Family Medicine, Taipei Medical University Hospital, Taipei 110301, Taiwan; 4 Department of Family Medicine, School of Medicine, College of Medicine, Taipei Medical University, Taipei 110301, Taiwan; 5 Nutrition Research Center, Taipei Medical University Hospital, Taipei 110301, Taiwan

**Keywords:** geriatric depression, bile acid, n-3 polyunsaturated fatty acid, brain−gut−microbiome axis, cognitive impairment

## Abstract

The brain−gut−microbiome (BGM) axis affects host bioinformation. N-3 polyunsaturated fatty acids (PUFAs) alleviate cognitive impairment and depression in older adults. This study investigated altered microbiota−bile acid signalling as a potential mechanism linking fish oil-induced gut changes in microbiota to alleviate psychological symptoms. Sprague Dawley rats were fed a fish oil diet and administered D-galactose combined with chronic unpredictable mild stress (CUMS) to simulate geriatric depression. The cognitive function, psychological symptoms, microbiota compositions, and faecal bile acid profiles of the rats were assessed thereafter. A correlation analysis was conducted to determine whether the fish oil-induced alteration of the rats’ microbiota and bile acid profiles affected the rats’ behaviour. D-galactose and CUMS resulted in lower concentrations of Firmicutes, significantly altered bile acid profiles, and abnormal neurobehaviours. Fish oil intake alleviated the rats’ emotional symptoms and increased the abundance of Bacteroidetes, Prevotellaceae, Marinifilaceae, and *Bacteroidesuniformis*. It also elevated the concentrations of primary bile acids and taurine-conjugated bile acids in the rats’ faeces. The rats’ taurine-conjugated bile acid levels were significantly correlated with their behavioural outcomes. In short, fish oil intake may alleviate psychological symptoms by altering the microbial metabolites involved in the BGM axis, especially in the conjugation of bile acids.

## 1. Introduction

Considerable evidence of the association between human health and gut microbiota has been uncovered within the last decade [1]. Gut microbiota is essential for maintaining physiological homeostasis. Abnormal physical status (including obesity, type 2 diabetes mellitus, non-alcoholic fatty liver disease, and other neuropsychiatric diseases) are strongly associated with the gut microbiome [2]. However, the actual physical interactions among the gut microbiome, gut, and other organs remain poorly understood.

The signals produced by gut microbiota are connected, not only to neighbouring regions, but also to distant tissues and organs, including the brain (as the brain−gut−microbiome [BGM] axis), the liver (as the liver−gut−microbiome axis), and parts of the immune system (as the microbiota−gut−immune−glia axis) [3]. The BGM regulates the homeostasis of the central nervous system [4]. Dysbiosis affects the integrity of the gut, brain, and blood−brain barrier (BBB), resulting in neuropsychiatric behaviours in animals [5]. Impaired organ perfusion structures neither block the passage of harmful cells and bacteria-derived metabolites into organs from the blood nor transmit standard physiological signals to target organs. Bacteria-derived metabolites induce immediate immune responses, resulting in mental disruption and the subsequent development of psychological disorders, such as cognitive impairment and depression [6,7]. Although the role of gut microbiota in human health has been widely acknowledged, the effects of gut microbiota on physiological homeostasis and their underlying molecular mechanisms remain poorly understood.

One key aspect of the relationship between microbiota and their host is that both host- and microbiota-derived enzymes regulate intravenous metabolites and affect the host in various manners [8,9]. Microbiota and the bioactive metabolites they produce regulate many physiological pathways and play a crucial role in maintaining human health. Studies have revealed that kynurenine-related metabolites and short-chain fatty acids are the potentially effective compounds of enormous microbiota metabolites [10,11]. Another important class of microbiota-derived metabolites is bile acids, which are produced mainly in the liver [12].

Bile acids are endogenously synthesised from cholesterol and stored in the gallbladder. They are released into the duodenum after food intake, reabsorbed in the ileum, and then recirculated to the liver via the hepatic portal vein in a process called “enterohepatic circulation”, which preserves more than 95% of the bile acid pool. Some classes of microbiota can deconjugate conjugated primary bile acid in reactions catalysed by bile acid hydrolase (BSH), which prevents bile reuptake through enterohepatic circulation. The microbial metabolism of bile acids leads to increased microbial diversity and, in general, a more hydrophobic bile acid pool, and facilitates faecal elimination of bile acids [13]. Secondary bile acids, which are microbially metabolised from unconjugated primary bile acids, are closely associated with the progression of cognitive impairment and Alzheimer’s disease (AD) [14]. By contrast, ursodeoxycholic acid (UDCA) has been demonstrated to exert neuroprotective effects by suppressing inflammation and rotenone-induced apoptosis in a rat model of Parkinson’s disease [15].

N-3 polyunsaturated fatty acids (PUFAs) are essential dietary nutrients and play central roles in learning, memory, and the regulation of the nervous and cardiovascular systems [16]. Chen et al. reported that gut dysbiosis might contribute to the pathogenesis of AD by inducing PUFA-associated neuroinflammation [17]. PUFAs slowed the short-term progression of illness in mice with abnormal neuropsychiatric behaviours by indirectly elevating the release of neurotransmitters [18] and modulated lipid disorders in humans by increasing levels of glycine-conjugated hyodeoxycholic acid (a secondary bile acid with hypolipidemic properties) [19]. However, whether dysbiosis caused by unhealthy lipid-rich diets and gut microbiota-derived metabolites (such as secondary bile acids) results in neurobehavioural changes remains unclear. Therefore, we investigated whether different dietary oil interventions affected dysbiosis in a chronic unpredictable mild stress (CUMS)- and D-galactose (D-gal)-induced ageing model and explored the possible mechanisms underlying the lipids on intestinal dysbiosis and neuropsychiatric behaviours induced by CUMS. Considering the neuroprotective effects of n-3 PUFAs, we also analysed the associations between n-3 PUFAs and changes in bile acid profiles.

## 2. Experimental Section

### 2.1. Animals, Diets, and Experimental Design

Male Sprague Dawley rats (*n* = 30, 6 weeks old) were obtained from the animal facility of BioLASCO, Taipei, Taiwan. The rats were housed under controlled environmental conditions (temperature: 22 °C ± 2 °C, humidity: 60%, and a 12-h light−dark cycle [light 08:00 to 20:00]). The study was conducted in accordance with the institutional guidelines and the study protocol was approved by the Taipei Medical University Institutional Animal Care and Use Committee (Permit number: LAC-20160405). After a 2-week acclimation period, the rats were divided into six groups: normal control (N) group; D-gal group (ND group); D-gal with CUMS group (CD group); CD treated with imipramine (PCD) group; CD with fish oil diet (FCD) group, and CD with corn oil diet (CCD) group. Since imipramine (tricyclic antidepressant) is a well-known and effective medicine for depression, we treated rats with imipramine in the drinking water (20 mg/kg body weight) as a positive control (PCD group). The components of the diets were in accordance with the American Institute of Nutrition-93 M diet (AIN-93 M, MP Biomedicals, Irvine, CA, USA) with 4% (*w*/*w*) dietary oil adjusted for this study. The normal diet consisted of 4% (*w*/*w*) soybean oil, the fish oil diet consisted of 2% fish oil and 2% soybean oil, and the corn oil diet consisted of 2% corn oil and 2% soybean oil. The vitamin E-containing fish oil (with 18% eicosapentaenoic acid [EPA] and 12% docosahexaenoic acid [DHA]) was purchased from Chueh Hsin Corp. (New Taipei City, Taiwan). The soybean and corn oils were purchased from a local supermarket. The fatty acid compositions of the oils and the diet composition are listed in Table 1. All animals were provided with their assigned diets and water ad libitum. During the experiment, the food consumption and body weights of experimental rats were recorded.

**Figure 1:**
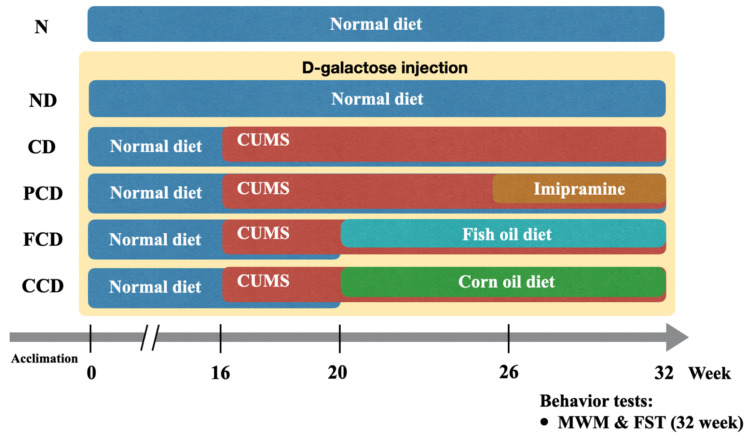
Flow chart of the experiment protocol. Rats were divided into six groups (*n* = 6). CUMS: chronic unpredictable mild stress; SPT: sucrose preference test; MWM: Morris water maze; FST: forced swimming test.

The experiment was conducted over 32 weeks (Figure 1). The D-gal injections (s.c. 500 mg/kg) were administered continually during the experimental period to every group except for the N group. The CUMS protocol was applied from week 16 until the end of the experiment. The fish oil and corn oil diets were provided to the FCD and CCD groups, respectively, from week 20 until the end of the experiment. Imipramine (Sigma-Aldrich, Carlsbad, CA, USA) was administered in the drinking water (20 mg/kg) to the PCD group daily from week 26 to week 32.

### 2.2. CUMS Model

The main objective of the CUMS model is to induce psychological stress rather than physical pain to simulate the pathological mechanism of depression in humans. We implemented the CUMS protocol by randomly exposing the rats (except those in the N and ND groups) to multiple stressors continually over a 16-week period (from week 16 to week 32). The rats were exposed to six of the following nine possible stressors each week: 30° cage tilt (12 h), food and water deprivation (24 h), damp sawdust (250 mL of water in their sawdust bedding, 24 h), lack of sawdust bedding (24 h), reversal of circadian rhythm, restraint in a plastic bag (1 h), limitation of living space (12 h), social stress (12 h), and cold swimming (10 °C, 1 h). None of the same stressors were applied for two consecutive days [20,21].

### 2.3. Detection of Biochemical Parameters and Inflammatory Cytokines

Plasma samples were collected via the abdominal aorta after finishing the FST immediately and stored for the following analysis with parameters. The fasting blood glucose (FBG), albumin (ALB), triglyceride (TG), total cholesterol (TC), low-density lipoprotein (LDL) cholesterol, and high-density lipoprotein (HDL) cholesterol, aspartate aminotransferase (AST), blood urea nitrogen (BUN), and creatinine (CRE) were measured in the plasma samples. The preceding parameters were measured using a UniCel DxC 800 clinical chemistry analyser (Beckman Coulter, Carlsbad, CA, USA). According to the manufacturers’ instructions, the level of corticosterone in the plasma was examined using a rat corticosterone ELISA kit (Abcam, Cambridge, UK).

The proinflammatory indicator’s tumour necrosis factor (TNF-α), interleukin-1β (IL-1β), and interleukin-6 (IL-6) were measured using plasma. The plasma TNF-α level was detected by a TNF-α enzyme-linked immunosorbent assay kit (Cat. No. 438207, BioLegend, San Diego, CA, USA). In addition, IL-1β and IL-6 levels were determined by an IL-1β Quantikine ELISA kit (Cat. No. RLB00, R&D Systems, Minneapolis, MN, USA) and IL-6 Quantikine ELISA kit (Cat. No. R R6000B, R&D Systems, USA), respectively.

### 2.4. Behavioural Tests

The forced swimming test (FST) is a behavioural despair model that can adequately predict an antidepressants’ efficacy. The FST was conducted following a previously reported protocol with slight modifications [22]. In the pre-test session, each rat was placed in a Plexiglas cylinder (height: 70 cm; diameter: 37 cm) containing 50 cm of water (24 °C ± 1 °C) for 15 min to induce the despair emotion and then dried with a towel and placed in a cage with an infrared lamp to recover. After 24 h, each rat performed under the same conditions for a 5-min probe trial. The experimental procedure was recorded with a camera (SONY, Tokyo, Japan). We analysed the videos using Forced Swim Scan 2.0 software (CleverSys, Reston, VA, USA) to detect the time of immobility (an indicator of despair), swimming, and struggling (including climbing, diving, and attempting to escape).

We evaluated the rats’ cognitive spatial learning and memory by conducting the Morris water maze (MWM) following the previously described research [23]. The apparatus used to perform the test consisted of a black-painted open circular pool (height: 100 cm, diameter: 150 cm) filled approximately halfway with RT water (20 °C ± 2 °C) and a hidden platform placed below the water surface. The apparatus was surrounded by extra maze cues placed on the room’s walls. Before the probe trial, the rats underwent training (four trials/day consecutively for three days). We measured each rat’s escape latency (time required to reach the platform) as a learning indicator during the training period. For the last day of the probe trial, the platform was eliminated, and each rat was forced to swim for 40 s. We used the time swam in the target quadrant as an indicator of memory in the probe trial. The whole test procedure was recorded and analysed using CineLyzer software (Plexon, Dallas, TX, USA).

### 2.5. Faecal Microbiota Analysis

Faecal samples were collected before the rats were killed and then stored at −80 °C immediately. We extracted the DNA from 200-mg faecal samples using the PowerSoil DNA Isolation Kit (Qiagen, Hilden, Germany) under the manufacturer’s instructions. We used the 16S amplicon sequencing method to amplify the 16s ribosomal RNA (rRNA) and an Illumina MiSeq system (San Diego, CA, USA) to sequence the 16s rRNA genes extracted from the faecal samples. We demultiplexed the raw data (Illumina CASAVA v1.8), merged the paired-end reads, and removed any chimaeras (UCHIME). We used UPARSE to cluster operational taxonomic units (OTU), with a similarity cut-off of 97%. The inferred amplicon sequence variants were subjected to taxonomy assignment using the SILVA database (v132), with a minimum bootstrap confidence of 80%. We estimated the community diversity on the basis of the normalised reads by using the Shannon−Wiener diversity index and Simpson’s diversity index. We conducted partial least squares discriminant analysis (PLS-DA) and permutational multiple analysis of variance (MANOVA) to identify significant differences between samples.

### 2.6. Microbiota-Derived Bile Acid Analysis

We extracted 25 mg of frozen faeces using 1 mL of bile acid extraction reagent and following the instructions (BIOTOOLS, Taipei, Taiwan). After the extraction procedure, we transferred the supernatant for bile acid analysis using the Waters ultra-high-performance liquid chromatography system coupled with a Waters Xevo tandem quadrupole mass spectrometer (TQ-S MS, Waters, Milford, MA, USA). Chromatographic separation was performed using Waters ACQUITY BEH C8 column (1.7 μm, 2.1 mm × 100 mm). The column temperature was maintained at 60 °C. Mobile phase A was 10% acetonitrile, adding 0.01% formic acid for optimised parameters and mobile phase B was isopropanol/acetonitrile (50:50, *v*/*v*), adding 0.01% formic acid. The following analysis was conducted using a Waters Xevo TQ-S system (positive ESI method). The capillary voltage was set to 1.5 kV. The desolvation gas flow rate and the cone gas flow rate were set to 1000 and 150 L/h, respectively. The desolvation and source temperatures were set to 600 °C and 150 °C, respectively. A quality control (QC) and mixed QC samples (a mixture of all samples) were run after every ten samples.

### 2.7. Statistical Analysis

We conducted our statistical analyses using Prism 9.1.1 (GraphPad Software, La Jolla, CA, USA). The data were subjected to one-way analysis of variance, followed by Tukey’s post hoc test. R Studio 4.1.2 (R Studio Software, Boston, MA, USA) was used to conduct the permutational MANOVA (ADONIS) and create heatmaps. We identified correlations between pairs of microbial features through Spearman’s correlation analysis, followed by a Kruskal−Wallis test. The correlations among microbiota abundance, bile acid levels, and neurobehavioural outcomes were analysed using Pearson’s correlation coefficient.

## 3. Results

### 3.1. Biochemical and Inflammatory Status

We found significant decreases in plasma TG and LDLc levels in the FCD group compared with the control group. Regarding the inflammatory status, the plasma levels of proinflammatory cytokines, such as TNF-α, IL-1β, and IL-6, exhibited a significant increase in the ND and CD groups compared to the N group. The proinflammatory effects of FCD on plasma TNF-α and IL-1β (but not IL-6) levels were significantly restored by fish oil (Table 2). In addition, results also demonstrated that the rats under CUMS secreted more corticosterone than the group N rats. It showed that the CUMS might cause the corticosterone overexpression and the fish oil intervention can significantly ameliorate it.

### 3.2. Between-Group Comparison of Microbial Diversity

**Figure 2:**
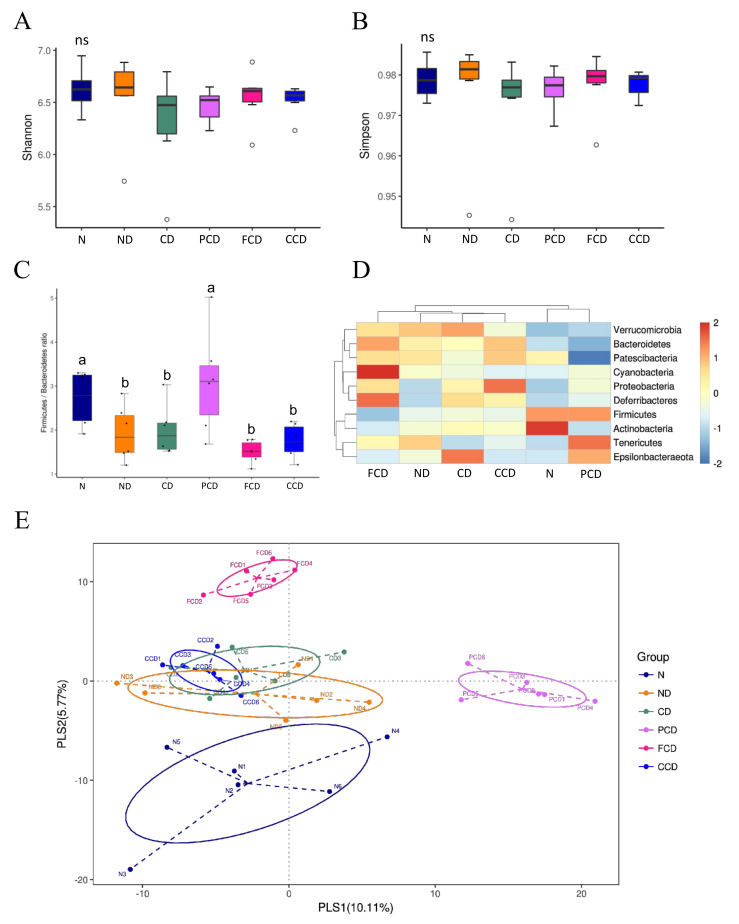
Diversity analysis of microbiota compositions. (**A**) Shannon−Wiener diversity index; (**B**) Simpson’s diversity index; (**C**) Firmicutes/Bacteroidetes (F/B) ratio; (**D**) Heatmap of phylum abundance (**E**) Differences among groups were visualised through partial least squares discriminant analysis (*n* = 6). ns = no significant difference. Different superscript letters indicated significant differences among groups at *p* < 0.05.

We analysed the Firmicutes/Bacteroidetes (F/B) ratios of the groups to identify potential alterations in the abundances of two dominant phyla in rats exposed to CUMS and fed different dietary lipids, and we used the Simpson’s diversity index and Shannon−Wiener diversity index to obtain additional information regarding microbiota composition. The results of 16s rRNA sequencing analysis revealed that the microbiota compositions of the rats were affected mainly by D-gal, fish oil, and pharmaceutical treatment. First, the groups receiving D-gal injections had significantly lower F/B ratios than the N group. CUMS did not cause significant changes in the rats’ microbiota in terms of F/B ratio or PLS-DA results (Figure 2C,E). The effect of fish oil intake (in the FCD group) on the microbiota composition was reflected in the PLS-DA results, which showed that the FCD group composition was significantly different from that of the CD and CCD groups. Furthermore, fish oil intervention influenced the gut microbiome composition. Specifically, the FCD group had a lower abundance of Firmicutes, but a higher abundance of Cyanobacteria, Bacteroidetes, and Deferribacteres compared to other groups. Although with fewer Firmicutes and more Bacteroidetes, the F/B ratio of group FCD was not significantly different from the CD and CCD groups (Figure 2C).

### 3.3. Between-Group Comparison of Microbiota Composition

**Figure 3:**
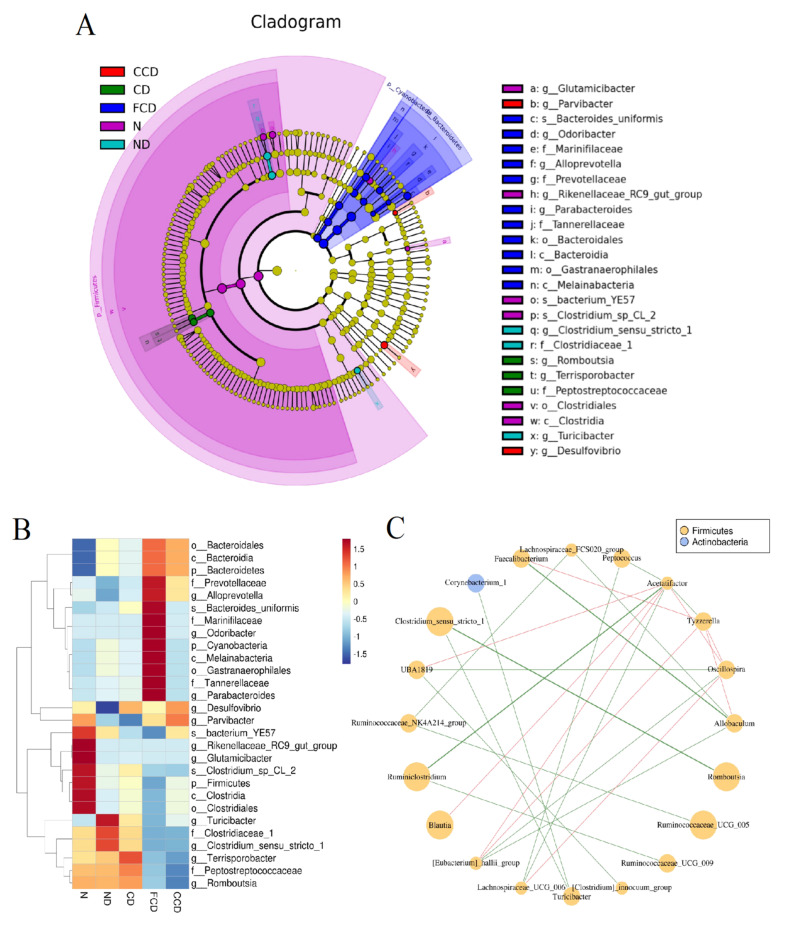
Linear discriminant analysis effect size analysis of microbiota compositions and network analysis of taxa. (**A**) Cladogram of microbiota compositions excluding the PCD group; (**B**) Heatmap of microbiota relative abundance; (**C**) Spearman’s correlation analysis of microbiota taxa, *r* cutoff = 0.7. Green lines indicate positive correlations. Red lines indicate negative correlations (*n* = 6).

According to the linear discriminant analysis effect size (LEfSe) results, the N group had the highest abundance of Firmicutes, including those of the genera *Glutamicibacter* and *Rikenellaceae_RC9_gut_group* and the species *Bacterium_YE57* and *Clostridium_sp_CL_2.* Compared with the N group, the ND group had a higher abundance of Clostridiaceae_1, *Turicibacter*, and *Clostridium_sensu_stricto_1* and a lower abundance of *Desulfovibrio*. However, the microbiota compositions of the CD group and the ND group did not differ significantly, indicating that CUMS did not significantly affect the gut microbiota of the rats. In addition, fish oil, which contains high concentrations of n-3 PUFAs, exerts strong effects on microbiota composition. The 12-week fish oil diet transformed the microbiota compositions of the rats in the FCD group, especially by reducing the abundance of Firmicutes. The abundance of Clostridiaceae_1, Peptostreptococcaceae, the genus *Clostridium_sensu_stricto_1*, *Turicibacter*, and *Bacterium_YE57* also decreased significantly in the FCD group, whereas the abundance of Prevotellaceae, Marinifilaceae, Tannerellaceae, *Alloprevotella, Odoribacter*, *Parabacteroides*, and *Bacteroides_uniformis* increased considerably. After the rats in the CCD group consumed the corn oil diet for 12 weeks, the abundance of *Bacteroidetes* in the CCD group was significantly lower than that in the N group. The abundance of *Romboutsia* and Peptostreptococcaceae also decreased in the CCD group. The abundance of *Desulfovibrio* and *Parvibacter* increased in all the groups. However, the PLS-DA revealed no significant changes in the microbiota composition of the CCD group (Figure 3A,B). Because of the various etiological factors involved, the data reflecting the effect of the medicine on gut microbiota (i.e., the data from the PCD group) were excluded from the microbiota analysis (Figure 3).

Spearman’s correlation analysis produced some interesting results. Several significant correlations between different types of microbiota were identified. For example, when we set the correlation coefficient threshold to 0.7, we observed that the abundance of *Faecalibacterium* was negatively correlated with that of *Tyzzerella,* suggesting that microbiota can affect each other. When we increased the threshold to 0.8, we observed strong positive correlations between the abundance of *Faecalibacterium* and *Allobaculum* and between the abundance of *Rombustia* and *Clostridium_sensu_stricto_1*. The abundance of Ruminiclostridium was also positively correlated with that of *Acetalifactor.* These findings may elucidate some mechanisms and interactions underlying microbial diversity in the gut.

### 3.4. Bile Acid Profile Changes Induced by Fish Oil

**Figure 4:**
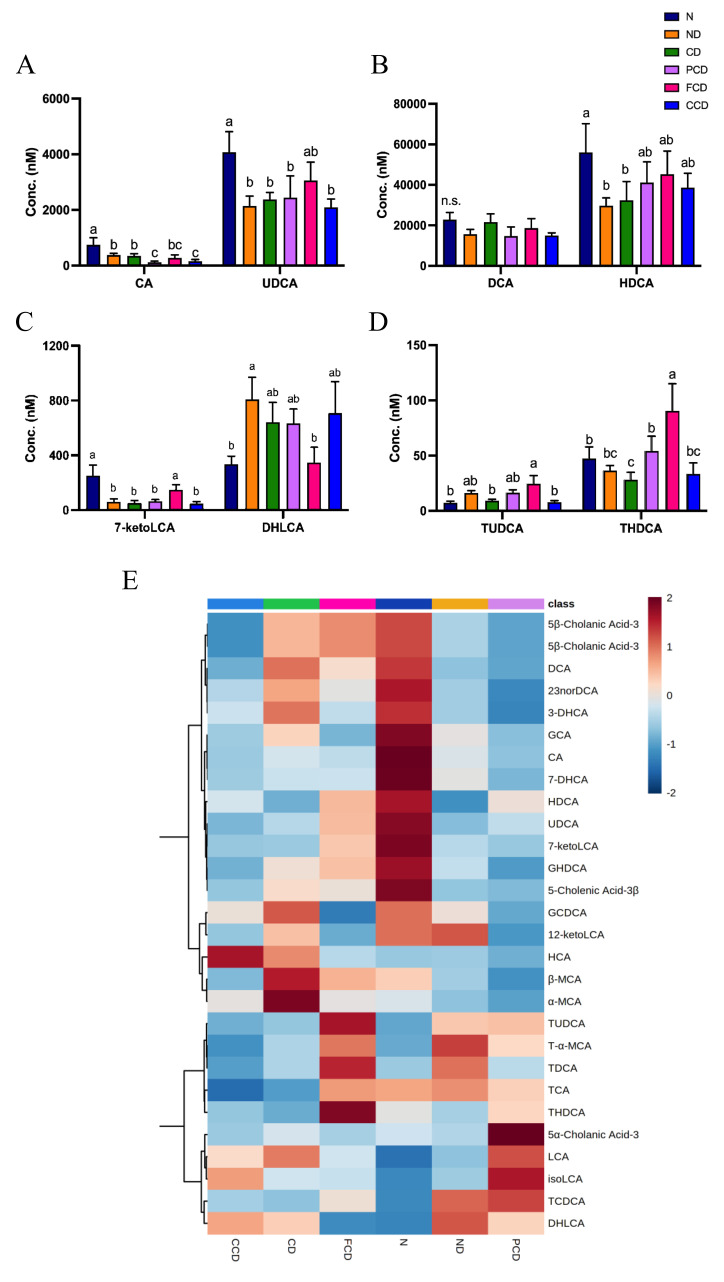
Bile acid profiles of rodent faecal samples. (**A**) Dominant primary bile acids in rodent faeces; (**B**) Dominant secondary bile acids in rodent faeces; (**C**) Levels of secondary bile acids 7−ketoLCA and DHLCA; (**D**) Levels of taurine-conjugated bile acids TUDCA and THDCA; (**E**) Heatmap of levels of 28 bile acids in each group. Data are expressed as means ± SDs (*n* = 6). Values with different superscript letters differ significantly (*p* < 0.05).

The levels of different bile acids in the rats’ faecal bile acid profiles, including the concentrations of the dominant primary bile acids (cholic acid [CA] and UDCA), are illustrated in Figure 4. The N group had the highest CA levels; both the PCD and CCD groups had CA levels lower than the N group. The N group had significantly higher UDCA levels than the ND, CD, PCD, and CCD groups (Figure 4A). In our analysis of the microbiota-derived secondary bile acids, deoxycholic acid (DCA) levels did not differ significantly among the groups. In contrast, the ND and CD groups had substantially lower hyodeoxycholic acid (HDCA) levels than the N group. We also analysed the secondary bile acids, 7-keto lithocholic acid (7-ketoLCA) and dehydrolithocholic acid (DHLCA). The levels of 7-ketoLCA in the N and FCD groups were significantly higher than those in the other groups. By contrast, the N and FCD groups had markedly lower DHLCA levels than the ND group (Figure 4B,C).

Bile acids were conjugated by taurine or glycine primarily in the liver and the conjugation process makes bile acids more hydrophilic and promotes bile acid excretion. In this study, we observed that the faecal samples of the FCD group had higher levels of taurine-conjugated bile acids, including taurine-ursodeoxycholic acid (TUDCA), taurine-hyodeoxycholic acid (THDCA), taurine-deoxycholic acid (TDCA), taurine-α muricholic acid, and taurine-CA (Figure 4D,E), compared to other groups.

### 3.5. Neuropsychiatric Behavioural Outcomes and Bile Acid Metabolites

**Figure 5:**
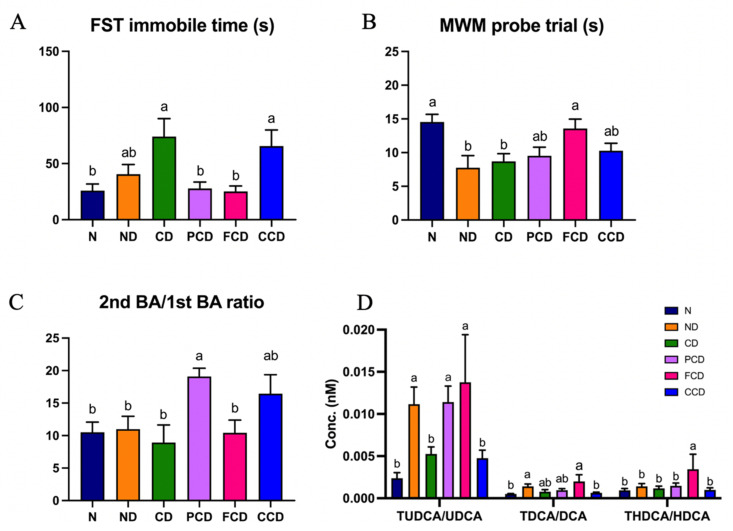
Neurobehavioural outcomes and results of bile acid metabolite analysis. (**A**) Time spent immobile during forced swim test; (**B**) Time spent in platform zone during Morris water maze trial; (**C**) Secondary/primary bile acid ratio in each group; (**D**) Proportions of taurine-conjugated bile acids in faecal samples. Data are expressed as means ± SDs (*n* = 6). Values with different superscript letters differ significantly (*p* < 0.05).

The FST is a conventional method of evaluating depressive-like behaviours in experimental animals, specifically through the assessment of the level of despair experienced by rodents during the test. In this study, compared with the N group, the CD and CCD groups spent significantly more time immobile during the FST (*p* < 0.05 for all), whereas the FCD and PCD groups did not. This indicates that imipramine and fish oil can prevent depressive-like behaviour in rodents. The MWM is often used to test the spatial memory function of rodents. The MWM probe trial results were used to determine whether the memories of the rats in different groups was impaired. The ND and CD groups spent less time in the platform zone than the N group (*p* < 0.05 for all), indicating that D-gal disrupted the memory function of the rats. Fish oil significantly improved the memory function of the FCD group (Figure 5A,B).

We also assessed the groups’ microbiota-derived metabolite ratios. The secondary/primary bile acid ratio of the PCD group was significantly higher than that of other groups (*p* < 0.05). We determined that the level of bile acid conjugation in the FCD group (measured using the THDCA/HDCA ratio) was higher than in the other groups. The TUDCA/UDCA and TDCA/DCA ratios did not differ significantly between the groups fed different types of oils; however, the ratios were higher in the FCD group. Interestingly, the proportion of taurine-conjugated BAs in the N group was substantially lower than that in the FCD group (*p* < 0.05; Figure 5C,D).

### 3.6. Correlations between Neurobehavioural Outcomes and Microbiota/Bile Acid Profile Characteristics

**Figure 6:**
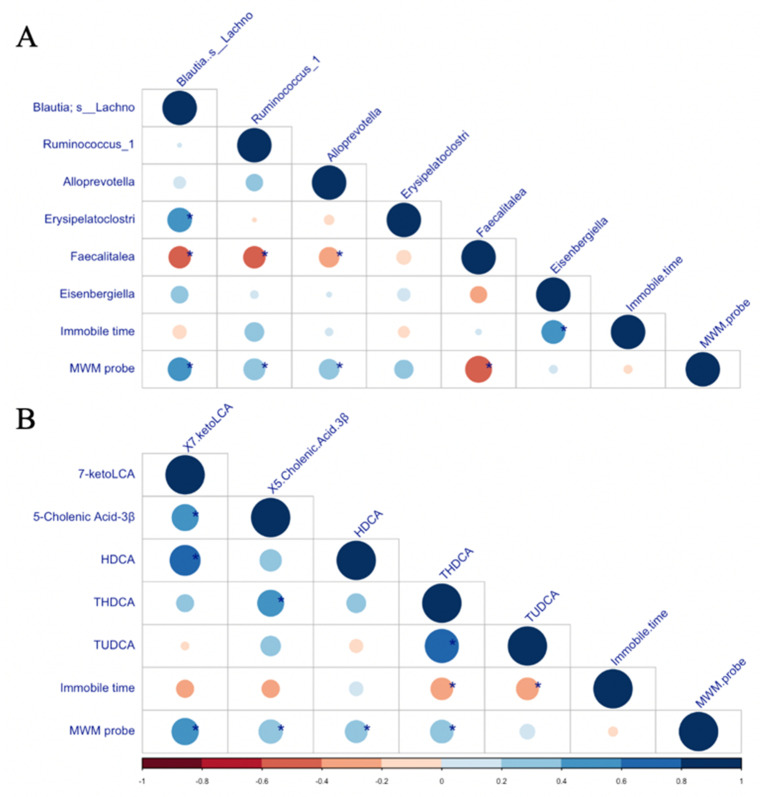
(**A**) The heatmap of Spearman’s correlation between the microbiota taxas and neurobehavioural test results. (**B**) The heatmap of Spearman’s correlation between the bile acids and neurobehavioural test results. Asterisk indicates significant differences between factors at *p* < 0.05.

The correlations among the selected microbiota, bile acids, and behavioural outcomes are presented in Figure 6. The depressive-like behavioural response (FST immobile time) was positively correlated with the abundance of *Eisenbergiella* and negatively correlated with THDAC and TUDAC levels. The cognitive behavioural response (MWM probe trial results) was positively correlated with the abundance of some microbiota (*Blautia; s__Lachnospiraceae_bacterium_609*, *Ruminococcus_1*, and *Alloprevotella*) as well as the levels of some bile acids (HDCA, 7- ketoLCA, 5-Cholenic acid-3β, and THDCA) and negatively correlated with the abundance of *Faecalitalea*.

## 4. Discussion

We administered D-gal and CUMS to simulate human geriatric depression. The D-gal-induced ageing model has been identified as an effective experimental ageing model in other studies [24,25]. We investigated how mimetic ageing combined with chronic physiological stress affects microbiota composition. We demonstrated that our approach simulated geriatric depression by evaluating cognitive impairment and depressive-like behaviour in rats through the MWM test and FST, respectively. According to the microbiota sequencing results, the microbiota compositions of the rats that received D-gal injections or were exposed to CUMS differed considerably from those of the N group. Studies have revealed that rodents fed a high-fat diet have an elevated abundance of Firmicutes and engage in depressive-like behaviours [26,27,28]. Unlike in other studies, the abundance of Firmicutes in the N group was significantly higher than that in the CD group. This difference indicated that a higher relative abundance of Firmicutes is not necessarily harmful to the host [29]. In clinical trials, Firmicutes and Bacteroidetes have been identified as the dominant phyla in young and older adults, respectively. Diminished microbiota diversity has also been observed in older adults. Although the dominant phylum has not been consistent across such trials, the level of microbial diversity did not differ significantly between the natural and mimetic ageing groups. In addition, one study revealed that naturally ageing mice had an increased abundance of Clostridiaceae [30,31]. Other clinical trials have revealed that the relative abundance of *Clostridium_sensu_stricto_1* is positively associated with grey matter volume in the hippocampi of older adults [32,33]. In this study, the LEfSe analysis revealed that the abundance of Clostridiaceae_1 and *Clostridium_sensu_stricto_1* in the ND group was higher than that in the N group. We also observed that the abundance of *Blautia* and *Lactobacillus* in the N group was higher than in the ND group. By contrast, the abundance of *Akkermansia*, a class of probiotics, in the ND group was higher than that in the N group (Figure S1). Further comparison of microbiota compositions in mimetic and natural ageing models is warranted.

Changes in the gut microbiota of patients with major depressive disorder have been frequently discussed. For example, Enterobacteriaceae and *Alistipes*, the abundances of which are positively associated with depression, have been identified as a potential biomarker for use in depression diagnostics [29,34,35,36]. However, few studies have explored the association between geriatric depression and microbiota because of the difficulty of recruiting suitable participants. A double-blind, randomised clinical trial revealed that the abundance of Prevotellaceae and *Eubacterium* decreased in patients with geriatric depression who received probiotics containing *Bifidobacterium bifidum BGN4* and *Bifidobacterium longum BORI* for 12 weeks [37]. Another placebo-controlled prospective pilot study revealed that the abundance of *Faecalibacterium, Agathobacter*, and *Roseburia* was associated with the treatment outcome of remission in geriatric depression [38]. In the present study, the CD group, which mimics geriatric depression symptoms, had higher abundances of *Faecalitalea* and *Akkermanisa* than the N group. In addition, the ND and CD groups had a higher abundance of Christensenellaceae and *Christensenellaceae_R_7_group* than the N group (Figure S2). The abundance of Christensenellaceae was reported to be negatively associated with the weight of visceral adipose tissue in a study of Italian older adults [39].

Accumulating evidence supports an association between n-3 PUFAs and microbiota composition. N-3 PUFAs are essential fatty acids for humans and have been determined to alleviate inflammation and neurobehavioural symptoms in other studies [40,41,42,43]. Researchers have proposed that n-3 PUFAs directly affect gut microbial diversity and alter the metabolic function of the host. One study of liver injury induced by chronic alcohol intake revealed that fish oil supplements could substantially affect gut microbiota and ameliorate liver injury [44]. Another study showed that fish oil intake alleviated recurrent obesity induced by a high-fat diet, reduced body weight, minimised net weight gain, and affected body fat distribution in mice. The microbiota compositions of the mice, especially in terms of the relative abundances of *Bacteroidetes*, Lachnospiraceae, and Bifidobacterium, responded to the rapid dietary changes between the fish oil and the high-fat diet [45]. In the present study, the FCD group had a higher abundance of Bacteroidetes than the CD group. The abundance of *Cyanobacteria* and *Deferribacteres* was elevated in the FCD group. According to the LEfSe results, fish oil intake resulted in a higher relative abundance of Prevotellaceae, Marinifilaceae, Tannerellaceae, *Alloprevotella, Odoribacter,* and *Parabacteroides.* Notably, the FCD group had the highest abundance of a unique species, *Bacteroides_uniformis*, which modulated immunoregulatory function and reduced the M1/M2 macrophage ratio in the gut and adipose tissue of mice fed a high-fat, high-fructose diet in another study [46]. Further analysis regarding the direct effects of fish oil on the gut microbiota was also conducted. The between-group analysis of the FCD and CD groups showed that the genus *Lachonospiraceae_NK4A136_group*, *Parabacteroides,* and *Ruminococcus_1* were higher in the FCD group. In contrast, the *Akkermansia*, known as one of the probiotics in humans, was lower in group CD than group FCD (Figure S3). Overall, few experiments (clinical or preclinical trials) have been conducted to evaluate the effects of fish oil on gut microbiota in patients with geriatric depression. Additional studies on this topic are warranted.

The BGM axis has received considerable attention. The direct and indirect pathways between the gut and the brain, including the vagus nerve and the signalling pathways of microbiota-derived metabolites, have been investigated in numerous experiments. Bile acid has been determined to modulate the immune system and influence the progression of neurodegeneration [47,48,49]. Bile acids are endogenous molecules synthesised from cholesterol in the liver (primary bile acids), and some are further metabolised by the gut microbiota in the intestine and colon (secondary bile acids) [13]. The Alzheimer’s Disease Neuroimaging Initiative, an ongoing large multicentre clinical study, has characterised the strong association between bile acid profiles and AD [14].

Some studies have reported that studying bile acid may be an effective approach to elucidate the effects of the BGM axis on metabolic function. A study by Dehkordi et al. revealed that older adults with AD have lower plasma levels of primary bile acids than cognitively normal older adults. By contrast, secondary bile acids, especially DCA and its glycine- and taurine-conjugated forms, were strongly associated with cognitive decline. The researchers obtained similar results by comparing the ratios between pairs of bile acids. Four ratios—DCA:CA, TDCA:DCA, GDCA:DCA, and GLCA:CDCA—were significantly associated with cognitive function and AD, with higher ratios of secondary to primary bile acids being strongly associated with poorer cognitive function. The study also revealed that higher plasma levels of CA in older adults with mild cognitive impairment reflect a lower probability of conversion to AD [14]. In the present study, the levels of dominant primary bile acids in the N group, including CA and UDCA, were higher than those in the mimetic ageing groups, with greater cognitive function in the MWM test, which is consistent with the results of other studies. However, no association between cognitive function and levels of dominant secondary bile acids, namely DCA and HDCA, was observed. The concentration of secondary bile acids was not lower than that in the N group. The secondary BA/primary BA ratios of the group did not differ significantly except for that of the PCD group, which indicates the tremendous effect of imipramine on the gut microbiota and bile acid profiles.

The effects of n-3 PUFAs on the rats’ bile acid profiles also provided interesting insights into the BGM axis. Although we did not observe any changes in the levels of major primary and secondary bile acids in the FCD group, we did observe other changes in the group’s bile acid profile. First, the 7-ketoLCA levels of the FCD group, as well as the N group, were significantly higher than those of the other groups, and ketoLCA levels were positively correlated with cognitive function, as evaluated in the MWM probe trial. In another study, 7-ketoLCA was identified as a key intermediate in UDCA synthesis [50]. The FCD group had the highest levels of UDCA, which was determined to have neuroprotective properties that delayed neurological decline and affects a patient’s risk of developing hepatic encephalopathy in a preclinical study [51]. UDCA was also discovered to function as a potential therapeutic agent for Parkinson’s disease by inhibiting leucine-rich repeat kinase 2 (LRRK2) activity and preventing subsequent neuron loss [52].

Second, the three ratios of taurine-conjugated bile acids (primary or secondary) and the levels of TUDCA and THDCA were elevated in the FCD group. These results indicate that the n-3 PUFAs may promote taurine conjugation with bile acids or inhibit the deconjugation of taurine-conjugated bile acids. TUDCA has been identified as a neuroprotective agent in various studies [51]. TUDCA can prevent the apoptosis of neurons induced by amyloid-β (Aβ), avoid the aggregation of Aβ and the phosphorylation of tau proteins, and ameliorate neuroinflammation in AD through the E2F-1/p53/Bax pathway [53,54]. TUDCA was also reported to alleviate LPS-induced acute neuroinflammation by reducing the effects of pro-inflammatory stimuli on glial cells [55]. Another study revealed that TUDCA (administered through i.p. injections) might exert an anti-inflammatory effect by activating TGR5 receptors and the transforming growth factor β (TGFβ) pathway and modulating microglia/macrophage conversion in the brains of mice [49]. In the present study, the FCD group, which exhibited greater cognitive function in the MWM trial and fewer depressive-like behaviours in the FST, had higher levels of TUDCA and THDCA, which was negatively correlated with time spent immobile in the FST. These results indicate that TUDCA exerts neuroprotective effects and alleviates symptoms of depression. We also observed that THDCA levels were significantly and positively correlated with performance in the MWM probe trial. Although no studies have discussed the neurological effects of THDCA, it is an intriguing alteration of the bile acid profile after feeding with n-3 PUFA.

Bile acid signalling in the gastrointestinal tract has been well characterised. Nevertheless, few studies have investigated the underlying mechanisms of bile acid signalling in the brain. In this study, we used UPLC-MS/MS to quantify the faecal levels of various bile acids and compare them with the gut microbiota compositions of rats through next-generation sequencing to elucidate the complex relationships among gut microbiota, bile acid profiles, and neurobehavioural outcomes. However, this study still has several limitations, including those related to the distinct bile acid profiles of rodents and humans and the lack of analysis of the relationship between BBB function and bile acid concentration in the brain. Nevertheless, we still identified some noteworthy targets of the BGM axis in this study. Additional studies must be conducted further to evaluate the direct or indirect effects of bile acids and elucidate their role in the BGM axis of other animal models.

## 5. Conclusions

In summary, this study provided evidence of changes in the gut microbiota and bile acid profiles of rats with geriatric depression. We also observed the specific effects of n-3 PUFA intake on the rats’ gut microbiota compositions and bile acid profiles and identified some notable correlations with favourable neurobehavioural outcomes. These findings may serve as a reference for future investigations of the psychological effects of the BGM axis.

## Figures and Tables

**Table 1:** The composition of diet in different groups.

Ingredients (g/kg Dry Matter)	Group		
	N, ND, CD, PCD	FCD	CCD
Casein	140.00	140.00	140.00
L-Cystine	1.80	1.80	1.80
Corn starch	465.69	465.69	465.69
Dextrin	155.00	155.00	155.00
Sucrose	100.00	100.00	100.00
Cellulose	50.00	50.00	50.00
Soybean oil	40.00	20.00	20.00
Corn oil	0	0	20.00
LA	0	0	11.2
Fish oil	0	20.00	0
Eicosapentaenoic acid (EPA)	0	3.60	0
Docosahexaenoic acid (DHA)	0	2.40	0
AIN-93M Mineral mix	35.00	35.00	35.00
AIN-93M Vitamin mix	10.00	10.00	10.00
Choline bitartrate	2.50	2.50	2.50
t-butylhydroquinone	0.01	0.01	0.01
**Fatty acid profiles (%)**			
C14:0	1.42	5.63	1.23
C16:0	13.05	18.02	10.41
C16:1 (n-7)	0.07	0.04	0.03
C18:0	4.87	7.97	6.22
C18:1 (n-9)	17.79	9.95	19.01
C18:2 (n-6)	52.71	28.74	59.52
C18:3 (n-3)	9.88	6.19	3.26
C20:1 (n-9)	N.D.	0.27	N.D.
C20:2 (n-6)	N.D.	0.38	N.D.
C20:4 (n-6)	0.01	4.08	0.01
C20:5 (n-3)	N.D.	9.63	N.D.
C22:5 (n-3)	0.18	1.46	0.08
C22:6 (n-3)	N.D.	5.98	N.D.
n-6/n-3 ratio	5.24	1.30	17.82

N: normal control group; ND: D-galactose injection group; CD: CUMS + D-galactose injection group; PCD: CD + imipramine-treated group; FCD: CD + fish oil diet group; CCD: CD + corn oil diet group. Fish oil: containing 30% n-3 PUFAs, EPA:DHA = 3:2.

**Table 2:** Effects of fish oil on biochemical parameters in ageing rats.

Biochemical Characteristics	Group					
	N	ND	CD	PCD	FCD	CCD
Body weight	702 ± 48.3 ^a^	639 ± 73.8 ^ab^	583 ± 71.3 ^bc^	522 ± 62.3 ^c^	585 ± 79.1 ^bc^	622 ± 56.2 ^ab^
FBG (mg/dL)	99 ± 6 ^ns^	105 ± 7	109 ± 5	103 ± 8	96 ± 7	102 ± 5
ALB (g/dL)	4.14 ± 0.07 ^ns^	4.03 ± 0.10	4.00 ± 0.08	4.13 ± 0.04	4.11 ± 0.06	4.02 ± 0.09
TG (mg/dL)	71.3 ± 2.8 ^a^	80.7 ± 7.3 ^a^	69.2 ± 5.9 ^a^	73.9 ± 3.9 ^a^	60.2 ± 3.6 ^b^	78.8 ± 5.5 ^a^
TC (mg/dL)	66.6 ± 3.2 ^b^	70.4 ± 4.3 ^b^	81.6 ± 5.7 ^a^	74.5 ± 6.5 ^b^	68.8 ± 7.1 ^b^	72.9 ± 10.5 ^b^
LDLc (mg/dL)	6.89 ± 0.91 ^a^	7.11 ± 0.58 ^a^	7.94 ± 1.12 ^a^	5.82 ± 0.55 ^ab^	5.52 ± 0.31 ^b^	6.56 ± 0.72 ^a^
HDLc (mg/dL)	23.1 ± 1.4 ^ns^	24.5 ± 2.2	25.0 ± 1.7	24.2 ± 1.5	19.8 ± 1.8	23.6 ± 1.4
AST (U/L)	87.2 ± 7.2 ^ns^	76.9 ± 7.1	77.4 ± 6.1	81.8 ± 5.2	79.6 ± 2.9	85.2 ± 5.3
BUN (mg/dL)	20.8 ± 1.6 ^a^	19.7 ± 1.3 ^a^	19.9 ± 0.8 ^a^	16.1 ± 1.0 ^b^	15.8 ± 1.3 ^b^	19.1 ± 0.8 ^a^
CRE (mg/dL)	0.60 ± 0.04 ^b^	0.66 ± 0.06 ^ab^	0.59 ± 0.05 ^b^	0.72 ± 0.06 ^a^	0.68 ± 0.10 ^a^	0.69 ± 0.06 ^a^
TNF-α (pg/mL)	41.9 ± 3.1 ^b^	56.3 ± 6.2 ^a^	69.3 ± 3.7 ^a^	40.7 ± 5.1 ^b^	44.3 ± 2.6 ^b^	61.7 ± 4.2 ^a^
IL-1β (pg/mL)	20.2 ± 2.4 ^b^	48.6 ± 8.2 ^a^	49.4 ± 4.3 ^a^	32.3 ± 2.7 ^b^	24.1 ± 5.2 ^b^	43.4 ± 2.5 ^a^
IL-6 (pg/mL)	40.2 ± 1.9 ^ns^	41.8 ± 2.6	41.0 ± 2.1	39.4 ± 2.9	36.5 ± 2.8	38.2 ± 2.3
Corticosterone (ng/mL)	135 ± 14 ^b^	265 ± 27 ^a^	205 ± 19 ^a^	187 ± 11 ^a^	161 ± 13 ^b^	200 ± 16 ^a^

Fasting blood glucose (FBG), albumin (ALB), plasma triglyceride (TG), total cholesterol (TC), low-density lipoprotein cholesterol (LDLc), high-density lipoprotein cholesterol (HDLc), aspartate aminotransferase (AST), blood urea nitrogen (BUN), creatinine (CRE), tumour necrosis factor-alpha (TNF-α), interleukin-1 beta (IL-1β), interleukin-6 (IL-6) levels and plasma corticosterone levels. Values are presented as the mean ± SD (*n* = 6). ns = no significant difference. Different subscript letters indicated significant differences among groups at *p* < 0.05. N: control group; ND: D-galactose injection group; CD: CUMS + D-galactose injection group; PCD: CD + imipramine-treated group; FCD: CD + fish oil diet group; CCD: CD + corn oil diet group.

## Data Availability

The data presented in this study are available on request from the corresponding author. The data are not publicly available due to their size.

## Supplementary Materials

The following supporting information can be downloaded at: https://www.mdpi.com/article/10.3390/biomedicines10071594/s1, Figure S1: Analysis of mean difference in relative abundance at the generic level between the N and ND groups; Figure S2: Analysis of mean difference in relative abundance at the generic level between the N and CD groups; Figure S3: Analysis of mean difference in relative abundance at the generic level between the FCD and CD groups.

## References

[1] Gentile C.L., Weir T.L. (2018). The gut microbiota at the intersection of diet and human health. Science.

[2] Grüner N., Mattner J. (2021). Bile Acids and Microbiota: Multifaceted and Versatile Regulators of the Liver–Gut Axis. Int. J. Mol. Sci..

[3] Rudzki L., Maes M. (2020). The Microbiota-Gut-Immune-Glia (MGIG) Axis in Major Depression. Mol. Neurobiol..

[4] Wiley N.C., Dinan T.G., Ross R.P., Stanton C., Clarke G., Cryan J.F. (2017). The microbiota-gut-brain axis as a key regulator of neural function and the stress response: Implications for human and animal health. J. Anim. Sci..

[5] Lai W.-D., Tung T.-H., Teng C.-Y., Chang C.-H., Chen Y.-C., Huang H.-Y., Lee H.-C., Huang S.-Y. (2022). Fish oil ameliorates neuropsychiatric behaviors and gut dysbiosis by elevating selected microbiota–derived metabolites and tissue tight junctions in rats under chronic sleep deprivation. Food Funct..

[6] Braniste V., Al-Asmakh M., Kowal C., Anuar F., Abbaspour A., Tóth M., Korecka A., Bakocevic N., Ng L.G., Kundu P. (2014). The gut microbiota influences blood-brain barrier permeability in mice. Sci. Transl. Med..

[7] Kealy J., Greene C., Campbell M. (2018). Blood-brain barrier regulation in psychiatric disorders. Neurosci. Lett..

[8] Baier J., Gänsbauer M., Giessler C., Arnold H., Muske M., Schleicher U., Lukassen S., Ekici A.B., Rauh M., Daniel C. (2020). Arginase impedes the resolution of colitis by altering the microbiome and metabolome. J. Clin. Investig..

[9] Selwyn F.P., Csanaky I.L., Zhang Y., Klaassen C.D. (2015). Importance of Large Intestine in Regulating Bile Acids and Glucagon-Like Peptide-1 in Germ-Free Mice. Drug Metab. Dispos..

[10] Kimmel M., Jin W., Xia K., Lun K., Azcarate-Peril A., Plantinga A., Wu M., Ataei S., Rackers H., Carroll I. (2021). Metabolite trajectories across the perinatal period and mental health: A preliminary study of tryptophan-related metabolites, bile acids and microbial composition. Behav. Brain Res..

[11] Li Z., Lai J., Zhang P., Ding J., Jiang J., Liu C., Huang H., Zhen H., Xi C., Sun Y. (2022). Multi-omics analyses of serum metabolome, gut microbiome and brain function reveal dysregulated microbiota-gut-brain axis in bipolar depression. Mol. Psychiatry.

[12] Yoshimoto S., Loo T.M., Atarashi K., Kanda H., Sato S., Oyadomari S., Iwakura Y., Oshima K., Morita H., Hattori M. (2013). Obesity-induced gut microbial metabolite promotes liver cancer through senescence secretome. Nature.

[13] Wahlström A., Sayin S.I., Marschall H.-U., Bäckhed F. (2016). Intestinal Crosstalk between Bile Acids and Microbiota and Its Impact on Host Metabolism. Cell Metab..

[14] MahmoudianDehkordi S., Arnold M., Nho K., Ahmad S., Jia W., Xie G., Louie G., Kueider-Paisley A., Moseley M.A., Thompson J.W. (2019). Altered bile acid profile associates with cognitive impairment in Alzheimer’s disease—An emerging role for gut microbiome. Alzheimer’s Dement..

[15] Abdelkader N.F., Safar M.M., Salem H.A. (2014). Ursodeoxycholic Acid Ameliorates Apoptotic Cascade in the Rotenone Model of Parkinson’s Disease: Modulation of Mitochondrial Perturbations. Mol. Neurobiol..

[16] Su H.-M. (2010). Mechanisms of n-3 fatty acid-mediated development and maintenance of learning memory performance. J. Nutr. Biochem..

[17] Chen C., Liao J., Xia Y., Liu X., Jones R., Haran J., McCormick B., Sampson T.R., Alam A., Ye K. (2022). Gut microbiota regulate Alzheimer’s disease pathologies and cognitive disorders via PUFA-associated neuroinflammation. Gut.

[18] Su K.-P., Lai H.-C., Yang H.-T., Su W.-P., Peng C.-Y., Chang J.P.-C., Chang H.-C., Pariante C.M. (2014). Omega-3 Fatty Acids in the Prevention of Interferon-Alpha-Induced Depression: Results from a Randomized, Controlled Trial. Biol. Psychiatry.

[19] Saraswathi V., Heineman R., Alnouti Y., Shivaswamy V., DeSouza C.V. (2019). A combination of Omega-3 PUFAs and COX inhibitors: A novel strategy to manage obesity-linked dyslipidemia and adipose tissue inflammation. J. Diabetes its Complicat..

[20] García-Díaz D.F., Campion J., Milagro F.I., Lomba A., Marzo F., Martínez J.A. (2007). Chronic mild stress induces variations in locomotive behavior and metabolic rates in high fat fed rats. J. Physiol. Biochem..

[21] Willner P. (2017). The chronic mild stress (CMS) model of depression: History, evaluation and usage. Neurobiol. Stress.

[22] Contreras C.M., Chacón L., Rodríguez-Landa J.F., Bernal-Morales B., Gutiérrez-García A.G., Saavedra M. (2004). Spontaneous firing rate of lateral septal neurons decreases after forced swimming test in Wistar rat. Prog. Neuro-Psychopharmacol. Biol. Psychiatry.

[23] Vorhees C.V., Williams M. (2006). Morris water maze: Procedures for assessing spatial and related forms of learning and memory. Nat. Protoc..

[24] Yanar K., Simsek B., Atukeren P., Aydin S., Cakatay U. (2019). Is D-Galactose a Useful Agent for Accelerated Aging Model of Gastrocnemius and Soleus Muscle of Sprague-Dawley Rats?. Rejuvenation Res..

[25] Azman K.F., Zakaria R. (2019). d-Galactose-induced accelerated aging model: An overview. Biogerontology.

[26] Bruce-Keller A.J., Salbaum J.M., Luo M., Blanchard E., Taylor C.M., Welsh D.A., Berthoud H.-R. (2014). Obese-type Gut Microbiota Induce Neurobehavioral Changes in the Absence of Obesity. Biol. Psychiatry.

[27] Dutheil S., Ota K.T., Wohleb E.S., Rasmussen K., Duman R.S. (2016). High-fat diet induced anxiety and anhedonia: Impact on brain homeostasis and inflammation. Neuropsychopharmacology.

[28] Zhao L., Zhang Q., Ma W., Tian F., Shen H., Zhou M. (2017). A combination of quercetin and resveratrol reduces obesity in high-fat diet-fed rats by modulation of gut microbiota. Food Funct..

[29] Jiang H., Ling Z., Zhang Y., Mao H., Ma Z., Yin Y., Wang W., Tang W., Tan Z., Shi J. (2015). Altered fecal microbiota composition in patients with major depressive disorder. Brain Behav. Immun..

[30] O’Toole P.W., Jeffery I.B. (2015). Gut microbiota and aging. Science.

[31] Kumar M., Babaei P., Ji B., Nielsen J. (2016). Human gut microbiota and healthy aging: Recent developments and future prospective. Nutr. Heal. Aging.

[32] Lee S.M., Milillo M.M., Krause-Sorio B., Siddarth P., Kilpatrick L., Narr K.L., Jacobs J.P., Lavretsky H. (2022). Gut Microbiome Diversity and Abundance Correlate with Gray Matter Volume (GMV) in Older Adults with Depression. Int. J. Environ. Res. Public Heal..

[33] Scott K.A., Ida M., Peterson V.L., Prenderville J.A., Moloney G.M., Izumo T., Murphy K., Murphy A., Ross R.P., Stanton C. (2017). Revisiting Metchnikoff: Age-related alterations in microbiota-gut-brain axis in the mouse. Brain Behav. Immun..

[34] Parker B.J., Wearsch P.A., Veloo A.C.M., Rodriguez-Palacios A. (2020). The Genus Alistipes: Gut Bacteria with Emerging Implications to Inflammation, Cancer, and Mental Health. Front. Immunol..

[35] Naseribafrouei A., Hestad K., Avershina E., Sekelja M., Linløkken A., Wilson R., Rudi K. (2014). Correlation between the human fecal microbiota and depression. Neurogastroenterol. Motil..

[36] Simpson C.A., Diaz-Arteche C., Eliby D., Schwartz O.S., Simmons J.G., Cowan C.S. (2020). The gut microbiota in anxiety and depression – A systematic review. Clin. Psychol. Rev..

[37] Kim C.-S., Cha L., Sim M., Jung S., Chun W.Y., Baik H.W., Shin D.-M. (2020). Probiotic Supplementation Improves Cognitive Function and Mood with Changes in Gut Microbiota in Community-Dwelling Older Adults: A Randomized, Double-Blind, Placebo-Controlled, Multicenter Trial. J. Gerontol. Ser. A.

[38] Lee S.M., Dong T.S., Krause-Sorio B., Siddarth P., Milillo M.M., Lagishetty V., Datta T., Aguilar-Faustino Y., Jacobs J.P., Lavretsky H. (2021). The intestinal microbiota as a predictor for antidepressant treatment outcome in geriatric depression: A prospective pilot study. Int. Psychogeriatr..

[39] Tavella T., Rampelli S., Guidarelli G., Bazzocchi A., Gasperini C., Pujos-Guillot E., Comte B., Barone M., Biagi E., Candela M. (2021). Elevated gut microbiome abundance of Christensenellaceae, Porphyromonadaceae and Rikenellaceae is associated with reduced visceral adipose tissue and healthier metabolic profile in Italian elderly. Gut Microbes..

[40] Tung T.-H., Nguyen N.T.K., Huang S.-Y. (2021). New Insights into Depressive Disorder with Respect to Low-Grade Inflammation and Fish Oil Intake. J. Oleo Sci..

[41] Joffre C., Rey C., Layé S. (2019). N-3 Polyunsaturated Fatty Acids and the Resolution of Neuroinflammation. Front. Pharmacol..

[42] Guo Y.R., Lee H.C., Lo Y.C., Yu S.C., Huang S.Y. (2018). n-3 polyunsaturated fatty acids prevent d-galactose-induced cognitive deficits in prediabetic rats. Food Funct..

[43] Su K.-P., Huang S.-Y., Chiu C.-C., Shen W.W. (2003). Omega-3 fatty acids in major depressive disorder: A preliminary double-blind, placebo-controlled trial. Eur. Neuropsychopharmacol..

[44] Chen Y.-L., Shirakawa H., Lu N.-S., Peng H.-C., Xiao Q., Yang S.-C. (2020). Impacts of fish oil on the gut microbiota of rats with alcoholic liver damage. J. Nutr. Biochem..

[45] Qin N., Song G., Ren X., Zhang L., Gao J., Xia X., Zhu B. (2020). Fish oil extracted from *Coregonus peled* improves obese phenotype and changes gut microbiota in a high-fat diet-induced mouse model of recurrent obesity. Food Funct..

[46] Fabersani E., Portune K., Campillo I., López-Almela I., la Paz S.M.-D., Romaní-Pérez M., Benítez-Páez A., Sanz Y. (2021). Bacteroides uniformis CECT 7771 alleviates inflammation within the gut-adipose tissue axis involving TLR5 signaling in obese mice. Sci. Rep..

[47] Zhao Y., Jaber V., Lukiw W.J. (2017). Secretory Products of the Human GI Tract Microbiome and Their Potential Impact on Alzheimer’s Disease (AD): Detection of Lipopolysaccharide (LPS) in AD Hippocampus. Front. Cell. Infect. Microbiol..

[48] Nunes A.F., Amaral J.D., Lo A.C., Fonseca M.B., Viana R.J., Callaerts-Vegh Z., D’Hooge R., Rodrigues C.M. (2012). TUDCA, a bile acid, attenuates amyloid precursor protein processing and amyloid-β deposition in APP/PS1 mice. Mol. Neurobiol..

[49] Yanguas-Casás N., Barreda-Manso M.A., Pérez-Rial S., Nieto–Sampedro M., Romero-Ramírez L. (2016). TGFβ Contributes to the Anti-inflammatory Effects of Tauroursodeoxycholic Acid on an Animal Model of Acute Neuroinflammation. Mol. Neurobiol..

[50] Huang B., Zhao Q., Zhou J.-H., Xu G. (2019). Enhanced activity and substrate tolerance of 7α-hydroxysteroid dehydrogenase by directed evolution for 7-ketolithocholic acid production. Appl. Microbiol. Biotechnol..

[51] McMillin M., Frampton G., Quinn M., Ashfaq S., de los Santos M., Grant S., DeMorrow S. (2016). Bile Acid Signaling Is Involved in the Neurological Decline in a Murine Model of Acute Liver Failure. Am. J. Pathol..

[52] Mortiboys H., Furmston R., Bronstad G., Aasly J., Elliott C., Bandmann O. (2015). UDCA exerts beneficial effect on mitochondrial dysfunction in LRRK2(G2019S) carriers and in vivo. Neurology.

[53] Ramalho R.M., Ribeiro P.S., Solá S., Castro R.E., Steer C.J., Rodrigues C.M. (2004). Inhibition of the E2F-1/p53/Bax pathway by tauroursodeoxycholic acid in amyloid beta-peptide-induced apoptosis of PC12 cells. J. Neurochemistry..

[54] Dionísio P., Amaral J.D., Ribeiro M.F., Lo A.C., D’Hooge R., Rodrigues C.M. (2015). Amyloid-β pathology is attenuated by tauroursodeoxycholic acid treatment in APP/PS1 mice after disease onset. Neurobiol. Aging.

[55] Yanguas-Casás N., Barreda-Manso M.A., Nieto-Sampedro M., Romero-Ramírez L. (2014). Tauroursodeoxycholic acid reduces glial cell activation in an animal model of acute neuroinflammation. J. Neuroinflammation.

