# Supplementary material for: N-3 PUFA Ameliorates the Gut Microbiota, Bile Acid Profiles, and Neuropsychiatric Behaviours in a Rat Model of Geriatric Depression
Source: Biomedicines. 2022 Jul 4;10(7):1594. doi: 10.3390/biomedicines10071594 (PMC9313093; doi:10.3390/biomedicines10071594)
Supplement: Supplementary file 1 [file biomedicines-10-01594-s001.zip › biomedicines-1775910-supplementary.pdf]

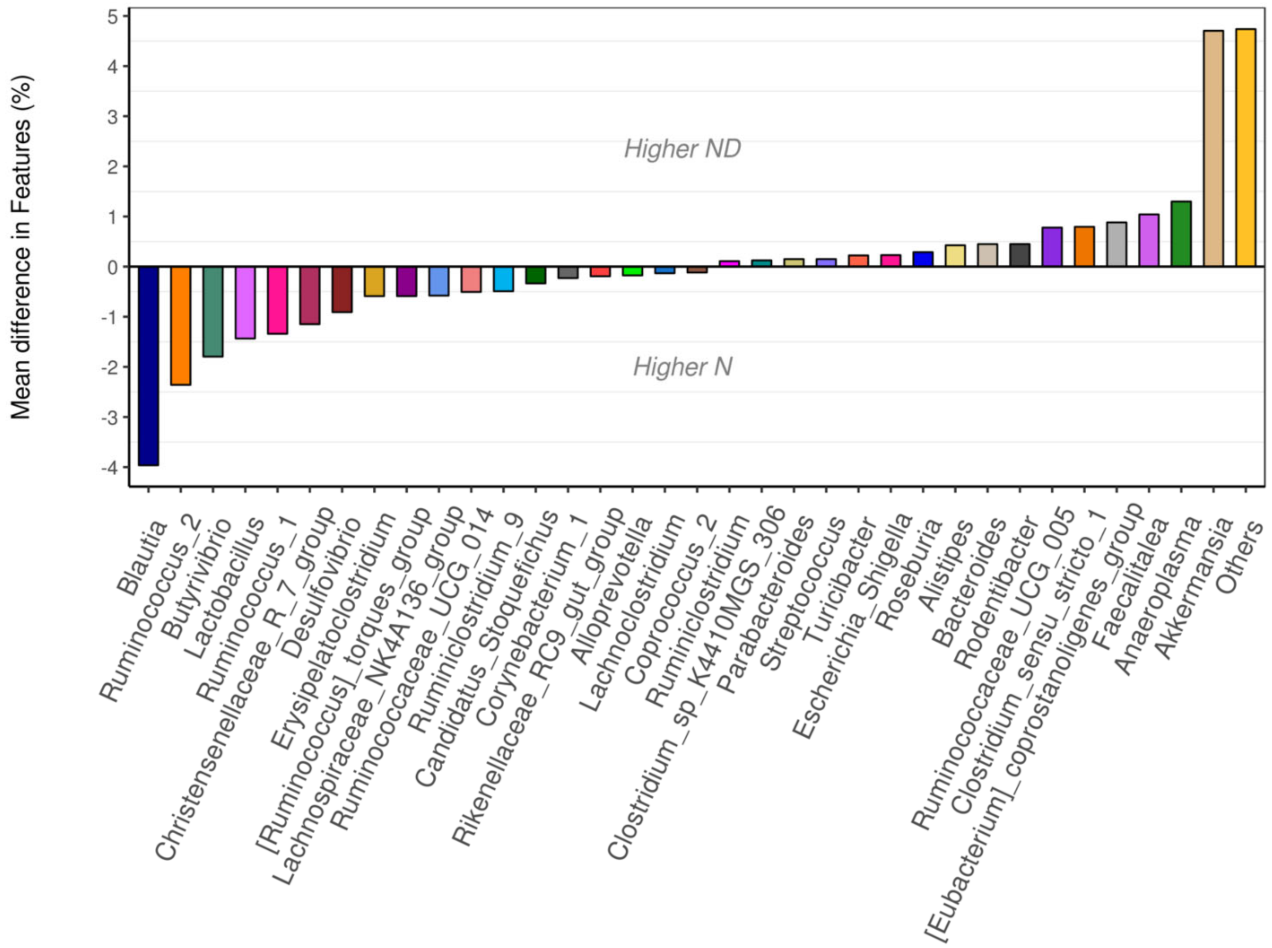

Figure S1. Analysis of mean difference in relative abundance at the generic level between the N and ND groups. Upper part indicates a higher percentage of genus relative abundance in the ND group. Lower part indicates a higher percentage of genus relative abundance in the N group.

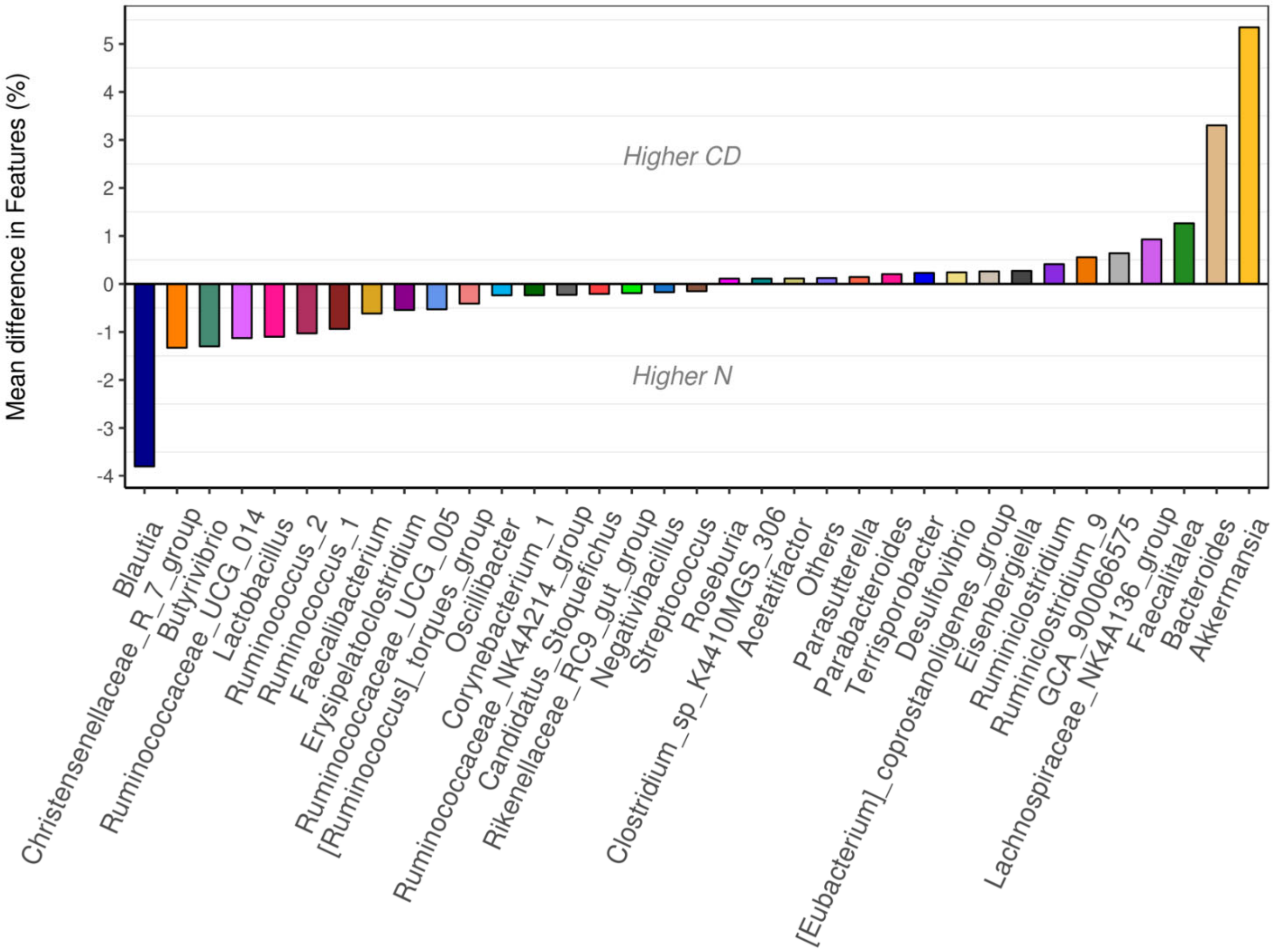

Figure S2. Analysis of mean difference in relative abundance at the generic level between the N and CD groups. Upper part indicates a higher percentage of genus relative abundance in the CD group. Lower part indicates a higher percentage of genus relative abundance in the N group.

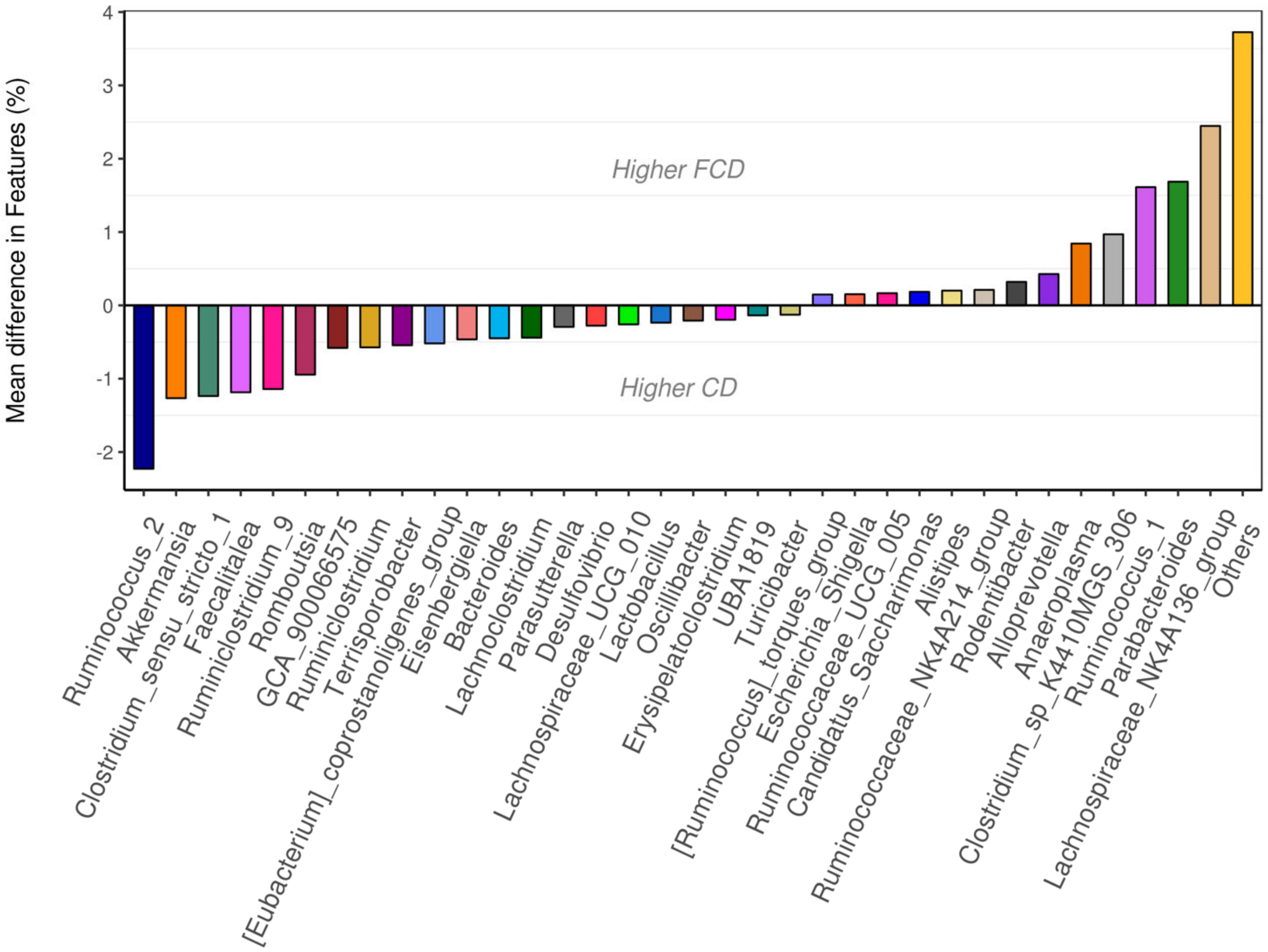

Figure S3. Analysis of mean difference in relative abundance at the generic level between the FCD and CD groups. Upper part indicates a higher percentage of genus relative abundance in the FCD group. Lower part indicates a higher percentage of genus relative abundance in the CD group.
